# Scaling parenting programs for early child development in four low- and middle-income countries

**DOI:** 10.3389/fpubh.2025.1604308

**Published:** 2025-10-28

**Authors:** Frances Aboud, Carina Omoeva, Rafael Contreras Gomez, Rachel Hatch, Ania Chaluda, Ksenija Krstic, Given Hapunda, Francis Sichimba, Karma Choden, Michael Tusiimi, Jill Popp

**Affiliations:** ^1^Department of Psychology, McGill University, Montreal, QC, Canada; ^2^Research and Evaluation Department, Washington, DC, United States; ^3^Department of Psychology, University of Belgrade, Belgrade, Serbia; ^4^Department of Psychology, Great East Road, University of Zambia, Lusaka, Zambia; ^5^Independent Researchers, Thimphu, Bhutan; ^6^College of Education, University of Rwanda, Rwaamagana, Rwanda; ^7^LEGO Foundation, Billund, Denmark

**Keywords:** early child development (ECD), parenting intervention, implementation evaluation, low- and middle income countries, indicators of successful scale

## Abstract

**Background:**

Although meta-analyses have demonstrated the value of parenting programs to promote child development in low- and middle-income countries, scaling them horizontally and vertically through the system has remained largely undocumented. This study examines the enablers and barriers to scaling parenting programs implemented by different organizations in four countries, namely Bhutan, Rwanda, Serbia, and Zambia.

**Method:**

An independent research and learning organization collected multi-method data from three sources, toward the end of a four-year period, to identify enablers and barriers of scale. The sources and method included: in-depth semi-structured interviews with two members of the technical resource teams (*n* = 8); phone surveys with a random sample of providers who delivered the program to caregivers (*n* = 529) along with in-depth interviews with a smaller number of providers (*n* = 44); and in-depth semi-structured interviews with key government stakeholders (*n* = 57). Content analysis was conducted to identify interviewees’ comments that reflected enablers and barriers to scale.

**Results:**

Findings are presented to address horizontal and vertical enablers and barriers in each of the four country programs. Regarding horizontal scale, the main enabler was an existing workforce who was quickly trained to deliver the program and who perceived a need within their communities. Expanding the reach of the programs also required advocacy to raise demand among community leaders and caregivers. Design features of the programs, such as curriculum, modality, and dosage, contributed to effective outcomes as a function of their adaptation to providers’ and caregivers’ experiences. The main enabler of vertical scale was adoption by the government, integration into the system, and engagement of multisectoral stakeholders. Based on final reflections of stakeholders, qualitative data were provided for eight indicators of successful scale: demand, reach, equity, and workforce (for horizontal scale); multisectorality, adoption, policy/finance, and integration (for vertical scale).

**Conclusion:**

Planning for scale needs to be done at the start by considering facilitative design features, selection of a workforce, and ownership by the government. Ongoing implementation research conducted with different stakeholders is needed to provide feedback for course-correction during the process of scale. Eight indicators can be used to evaluate the level of successful scale achieved by programs.

## 1 Introduction

Following publication of the World Health Organization’s recommendations for improving early childhood development (ECD) (1) and their subsequent ratification by the world’s ministers of health, governments in low- and middle-income countries (LMIC) have been seeking guidance on how to implement parenting programs for young children. Although still challenging, case studies [e.g., (2–5)] and a meta-analytic review (6) on key features of parenting program implementation are available. Less is known about how to scale parenting programs (7). One detailed case study of Brazil’s Criança Feliz (8) showed how a top-down approach led to gaps between municipal governments and caregivers. The present paper adds to this critical but scant literature by presenting independently collected data on four LMIC parenting programs as they transitioned to scale.

Scaling up is defined as the ‘deliberate efforts to increase the impact of successfully tested health innovations, so as to benefit more people and to foster policy and program development on a lasting basis’ (9). This definition, used for the ExpandNet framework, includes both horizontal (or geographic) reach and sustainable vertical integration within a health system. Understanding the nature of ECD parenting programs and the success or failure of their “deliberate efforts” is crucial for the field of ECD to support governments and organizations as they plan and proceed to scale their programs. The LEGO Foundation produced a document synthesizing learning from a series of meetings held with experts in the field on answers to critical questions about scaling up parenting programs in LMIC (10). Some of these focused on the program’s dosage and structure; others focused on the workforce, the demand (uptake) of the program, and its adoption by governments. As a result of our research into scaling up parenting programs, as reported here, we now provide some evidence-based answers to these challenging questions.

Within the ExpandNet framework, several key features of programs are thought to lend themselves to sustainable impact at scale. One is the design of the program (11), also known as intervention features (12) or as the innovation (9). Design refers to the selected curriculum, dosage, frequency, and delivery modality (home visits, group sessions, or clinic visits). These features should be aligned with current parenting practices in communities, knowledge of ECD held by the selected providers, and existing service provision. For example, among caregivers who provide insufficient responsive stimulation for child development, behavior change techniques such as demonstration, practice, and coaching are helpful: using three or more techniques resulted in a greater impact on child development and parenting practices in a recent review (6). For a workforce that is inexperienced in delivering messages on responsive stimulation, a structured curriculum to be followed at each contact ensures quality delivery (13). Finally, although home visits are more commonly used than group sessions (6), their reach might be less and possibly less cost-effective (14). Design features are usually selected in the initial phases but they might facilitate or hinder horizontal reach, quality of delivery, and effectiveness. Continuous feedback from implementation data allows for adjustments (2).

Because horizontal reach is a critical component of scale, implementation frameworks have identified the processes involved and what enables them (12, 15). The processes needed to expand horizontal reach include: providing outreach in remote areas, adapting to new communities with different needs, advocacy to raise demand, and training a new workforce in each district. Here, we focus on processes aimed at, and evidence for, increased reach, equity, engagement from the workforce, demand among caregivers, and advocacy to raise demand in communities.

Different programs vary in the goals they set for geographical coverage (16), but the actual number of people who participate (reach) depends also on the willingness of caregivers to attend and providers to deliver (17). While geographically smaller programs may use incentives and weekly reminders to encourage attendance at community groups (4), programs at scale eschew such costly additions and pay the price in lower attendance (16). Even home visiting programs at scale reported fewer than intended visits [e.g., 34% received no visits in Peru; (18)] due to provider issues such as lack of material and turnover (19).

The workforce is critical for reaching families with quality delivery; consequently their training and retention are central to scaling across communities. The choice of professional, para-professional or volunteer will influence the structure of the curriculum, length of training, regular supervision, and retention strategies. Professionals, such as nurses and health assistants, may have some expertise in child development and so need less training and supervision; however, their workload presents a challenge to taking on new responsibilities. Volunteers on the other hand may have little expertise, require more training and supervision, and an incentive (monetary or non-monetary) to retain them (20). Between these two status workers are para-professionals, for example community health workers (21) to whom many duties are shifted to reduce the heavy workload of professionals. Respect for the workforce may influence whether caregivers are quick or reluctant to uptake their services; furthermore, norms around parenting may result in caregivers trusting family more than professionals (15). Use of group vs. home visits may also be a preference among mothers who like the opportunity to learn from other parents (4). Finally, advocacy is a central feature of horizontal scaling as implementers aim to convince new communities of the benefits of receiving the service and so adapt the curriculum and delivery to their context.

Vertical scaling efforts are considerably more challenging as noted in the Criança Feliz case study (8). Working vertically within the health care system is essential to integrating a trained workforce and expanding the information system to track quality service delivery (22). At the same time, building capacity among government ministries and stakeholders is needed to sustain the political will to develop policy and ensure financial support. Raising demand throughout the system requires continuous advocacy along with evidence of effectiveness, especially during political turnover or instability. As noted in a recent curation of 13 scaled and transitioning-to-scale parenting programs in LMIC, Pethe (16) found that seven started at the top working with the Ministry of Health to initiate the parenting program. This top-down approach led to rapid adoption by the government but few attempts to get buy-in from communities or to provide initial evidence of effectiveness before scaling. For example, one of these top-down programs in Chile worked with the government from the start to create a policy and budget line and to institutionalize the program into law, with multisectoral responsibility from three ministries; an electronic database and tracking system provided accountability for deliverables (23). An associated program promoted parenting skills and later evaluated parent and child outcomes (24).

Some 71% of top-down programs had currently achieved scale. Of the other six, who used the bottom-up approach, only one-third had scaled; the others were transitioning to scale. They started with pilot programs in communities in order to select a suitable workforce and demonstrate effective gains in child and parent outcomes [e.g., (25, 26)]. Yet they typically encountered barriers when seeking government adoption and multisectoral partners. A critical component of a successfully scaled program—one that is arguably easier using a top-down approach—is the engagement of stakeholders from multiple sectors at the national, regional, and community levels, including academic institutions, civil society organizations, and ministries of health, education and social development (27). Thus, guidelines recommend assessing the initial readiness of the system to adopt a parenting program and building capacity as one transitions to scale (28).

The current paper describes the perspectives of multiple stakeholders engaged in the parenting programs implemented in four countries, with a focus on the final 2 years of scale-up. The four programs and their Resource Teams consisted of: Save the Children’s Prescription to Play in Bhutan (C4CD+), Boston College and FXB’s Sugira Muyango in Rwanda (SM), UNICEF’s Playful Parenting in Serbia (PP), and UNICEF’s Care for Child’s Healthy Growth and Development in Zambia (CCD). The programs began service in 2020, and during the final years of 2023–24 the perspectives of three groups of players were obtained by an independent research and learning group through surveys and in-depth interviews. The three players were: the Resource Team (implementing partners), the Workforce delivering the program, and Government Stakeholders. To determine the extent to which each had achieved scale in its final year, key informant interviews were conducted with government stakeholders in early 2025. This paper presents implementation evidence from four different parenting programs with a view to understanding enablers and barriers of moving to scale in these contexts. Evidence that reflects indicators of successful scale was used to identify the level achieved.

The overall question was: What implementation processes were involved as parenting programs in four countries worked toward and achieved scale?

Four specific research questions were:

Q1. How did design features contribute to scaling?

Q2. What implementation processes were used to expand horizontal reach; specifically, what enablers and barriers were expressed by the resource team, workforce and government stakeholders?

Q3. What processes were used to scale vertically from community to government; specifically, what enablers and barriers were expressed by the resource team and government stakeholders?

Q4. To what extent did programs finally achieve horizontal and vertical scale according to government stakeholders?

## 2 Materials and methods

### 2.1 Setting and participants

We conducted mixed-methods research on four parenting programs that were being implemented from mid-2020 to 2024 by different organizations in Bhutan, Rwanda, Serbia and Zambia. Data were collected annually by our research and learning group that included local co-investigators and local data collection firms.

The setting of each parenting program is best captured by statistics from the State of the World’s Children (29). We present the 2019 statistics because programs started in 2020 (see Table 1). All but Serbia were considered “least developed countries” so overall statistics for the 49 least developed countries are provided as a comparison; Bhutan rose to “full developing country” status in 2023. Although Rwanda had the lowest statistics on GDP per capita and literacy among adults, their health statistics were similar to or better than Zambia’s in terms of infant and child mortality, maternal mortality, water and sanitation, stunting and children’s diet. In Bhutan, Rwanda and Zambia, few children between 3 and 5 years of age were enrolled in an early childhood preschool program. All but Serbian parents lacked playthings and books in the home to provide stimulation for young children. The statistics indicate the need for parenting programs in these settings.

**Table 1:** Country statistics from state of the world’s children, 2019.

Indicator	Bhutan	Rwanda	Serbia	Zambia	Least Dev
Population ‘000	754	12,302	8,803	17,362	
% urban	41	17	56	44	34
GDP $ per capita	3,390	762	6,284	1,535	1,114
Gov’t budget %hlth	10	7.9	12.3	7.4	5.8
Primary school completed M, F	67, 71	48, 61	99, 100	73, 75	60, 60
Total Fertility rate	2.0	4.0	1.5	4.6	4.0
MMR 100,000LB	183	248	12	213	415
4 + antenatal, post-natal visits	85, 41	44, 43	94, −	56, 63	- -
Infant Mortality rate	25	27	5	40	46
Child Mortality rate	30	35	6	58	64
Stunting <5 yrs	34	37	6	40	32
Water, Sanitation	97, 69	58, 67	86, 98	60, 26	65, 34
ECD 3-5 yrs. enrolled	10	13	50	-	17
% Immunized (measles)	97	99	92	94	78
BF exclusive 6 m	51	87	13	70	51
Diet Diversity 4+	-	28	77	18	21
2 + Playthings 0–5y	52	30	75	-	46
3 + Books 0–5y	6	1	72	-	3

Statistics are given as a % unless otherwise indicated. Gov’t budget % hlth, % of government budget allocated to health. Total Fertility rate, mean number of children women will have. MMR, maternal mortality rate per 100,000 live births. Infant Mortality rate per 1,000 live births. Child mortality rate or the number of children per 1,000 born who will not reach their 5th birthday. Stunting, % of children less than 2SD of the median height for their age and sex. Water, Sanitation—% with improved source of water, safe disposal of feces. Dietary diversity, % of children under-5 eating 4 or more of 7 food categories daily.

#### 2.1.1 Study participants

Participants who provided data for the study are presented in Table 2, along with the method of data collection, sample sizes, and dates. The Resource Team (i.e., implementing partners) included one local and one international program director who provided technical support and oversaw the implementation of the parenting program before transferring to the government end-user. The workforce (also called providers) consisted of local men and women who were trained to deliver the parenting program to families in their village or municipality. Over 100 were randomly selected from lists of those who had been trained in the past year; they were interviewed with a structured survey guide by trained enumerators from a local data firm. Further, a purposive subsample of between 8 and 18 delivery agents in each country participated in in-depth interviews; they varied in gender and educational background. The same workers were interviewed annually to capture changes in their understanding of the program. Finally, a similar number of government stakeholders participated in in-depth interviews on an annual basis, with some changes if previously interviewed stakeholders were no longer engaged or available. They were selected with the help of the resource team to represent national and district-level people with a vested interest in the decision-making and activities of the program. For example, they were often connected to the Ministry of Health or other government sectors, and to local organizations or agencies who oversaw the workforce.

**Table 2:** Participants, measures, sample sizes, dates when administered.

Participant/measure	Bhutan Prescription to play	Rwanda Sugira Muryango	Serbia Playful parenting	Zambia Care for child’s healthy growth and development
1. Resource Team technical mgr in-depth interview				
Date of data collection	July 2023	May 2023	June 2023	August 2023
Sample size	*N* = 2	*N* = 2	*N* = 2	*N* = 2
2. Workforce Survey				
Date of data collection	April 2024	July 2024	Nov 2023	April 2024
Sample size	*N* = 150	*N* = 150	*N* = 111	*N* = 118
3. Workforce in-depth interview				
Date of data collection	June 2024	October 2024	Nov 2023	May 2024
Sample size	*N* = 8	*N* = 8	*N* = 10	*N* = 18
4. Government stakeholder interview				
Date of data collection	May 2024	April 2024	April 2024	March 2024
Sample size	*N* = 10	*N* = 9	*N* = 11	*N* = 7
Date of final interview	February 2025	February 2025	February 2025	February 2025
Sample size	*N* = 5	*N* = 4	*N* = 6	*N* = 5

#### 2.1.2 Ethical approvals

Ethical approval was obtained from the international research partner, an international university, and the local national ethics board in each country. Participants provided informed consent for each survey and interview. To maintain anonymity, details about the participants providing specific quotes are not given.

### 2.2 Methods of data collection and analysis

Many of the survey and interview questions were adapted from the ExpandNet worksheets (9) addressing the Innovation (design features of the program), the level of horizontal and vertical scale in the final year, and enablers and barriers to scale. They also touched on the plans and activities of the resource team to expand horizontally and vertically, perspectives of the workforce that identify enablers and challenges of meeting horizontal scale goals, and perspectives of government stakeholders regarding enablers and challenges of meeting both horizontal and vertical scale goals. Data were collected primarily in the final 2 years of the projects in order to focus on plans and activities directed toward achieving scale. Consequently, analyses of horizontal and vertical scale took place in two stages: first when the interview data were collected, transcribed, subjected to content analysis, and submitted to resource teams as reports. To make the findings of this content analysis transparent to the implementing resource teams, the extracted content remained close to the intention of the question and meaning of the answers. Second, these detailed reports were later curated by different researchers, organized by source and content, and revisited by the original analysts who added quotations from the transcripts. This two-step procedure for analyzing and curating the data is explained in greater detail in the next sections.

#### 2.2.1 Design features of programs

Research question #1 regarding design features of the program that might enable or challenge its scalability relied mainly on manuals outlining the curriculum and how the workforce was trained to deliver it. In-depth interviews with two key members of the four resource teams, one local and one international, were used to clarify design features and identify changes adopted usually to enhance horizontal scale. Content analysis was conducted on the relevant interview questions (30) by a local and an international researcher, and related evidence was sought from the program documents, specifically the manuals and guidelines used by providers. Revised manuals indicated where changes were made with the stated intention to reach more parents. The evidence of design showed little change across years.

#### 2.2.2 Enablers and barriers to horizontal scale

Research question #2 regarding horizontal scale relied on interviews with the resource teams, the workforce, and government stakeholders. The two key members of the resource team were initially asked about their plans for scaling horizontally, and in the penultimate year about the extent of horizontal scale, and challenges to reaching their goal. Workforce providers responded to questions as part of a structured phone survey conducted by trained enumerators from a local data firm. Reliability of answers was over 80%, according to follow-up interviews. Providers were asked about the number of families on their roster, the frequency of contact with families, the level of demand among families, and the respect they received from the community. In-depth interviews were repeated annually with a small number of providers to identify enablers and barriers to expanding their coverage of designated districts and a more nuanced understanding of whether families were adopting the parenting practices. These interviews were conducted by local investigators, who recorded, transcribed and translated the answers. Finally, government stakeholders were interviewed by local investigators using a semi-structured interview guide, asking for their perspective on the mechanisms of horizontal scale and the capacity of the workforce and resource team to reach scale. These interviews were recorded, transcribed and translated.

The analysis plan entailed a content analysis of answers to specific questions on strategies to achieve horizontal scale (30). For example, government stakeholders were asked the following questions about horizontal scale: Has demand for the program changed? How would you assess the capacity of the implementing team to expand their reach? What are the key capacity and resource gaps and how can they be remedied? The content analysis was conducted shortly after conducting interviews with each of the three sources. Both the local investigator and an international researcher conducted the content analysis by question and collected relevant quotes from respondents. Reports elaborating on answers to these questions were shared with and verified by the corresponding resource team in each country.

In preparation for this manuscript, the local researcher, the international researcher, and two additional researchers with experience in quantitative and qualitative research methods together clarified the definition of horizontal scale and its distinction from vertical scale. The detailed reports from the three sources in each country were perused by the two additional researchers with “fresh eyes,” and key statements extracted, organized into a table, and designated as an enabler or barrier. Responses along with quotes were organized by source and interview question and entered into a large table that designated each point as an enabler or barrier. The local and international researchers checked each entry and returned to the original transcripts to identify supportive quotes. Only entries that provided unique, non-redundant, and clear illustrations of horizontal scale were retained.

#### 2.2.3 Enablers and barriers to vertical scale

Research question #3 regarding vertical scale used questions from the semi-structured interview guide addressed to the resource team and government stakeholders. Interview questions concerning vertical scale asked specifically about the capacity and desire of the government to adopt the parenting program once the resource team’s activities and funding finished, about policy development, finances, integration of the providers into the government system for training and supervision, and the existence of an information system to track outputs and coverage. For example, questions asked to government stakeholders included: Do you feel you own this parenting program, and how is this manifest? How would you assess the capacity of the implementing team to transfer the program to the government? Do they have a feasible strategy for sustaining the program?

The analysis plan was similar to that for horizontal scale. Shortly after the interviews were conducted, the local investigator and an international researcher conducted the content analysis of transcripts by question and collected relevant quotes from participants. Reports elaborating on answers to these questions were shared with and verified by the corresponding resource team in each country. In preparation for this manuscript, two additional researchers who had been engaged in analyses were asked to go over the reports and extract information about vertical scale. We agreed on a definition of vertical scale. Responses along with quotes were organized by source and interview question and entered into a large table that designed each point as an enabler or barrier. The local and international researchers checked each entry and returned to the original transcripts to identify supportive quotes. Only entries that provided unique, non-redundant, and clear illustrations of vertical scale were reported.

#### 2.2.4 Evidence of level of achieved scale

Regarding research question #4, interviews were conducted in the final months with a limited number of key government stakeholders in each country to determine the final level of scale reached. At this time, donor funding was stopping or had stopped. Questions were asked specifically about the level of expansion to new communities and families (horizontal scale) and about specific activities to sustain the program through ownership, policy, financial support, an information management system, and capacity building in the government and multisector partners. Again, these interviews were conducted by local investigators who recorded, transcribed, and translated the interviews. Both local investigators and an international researcher identified quotes that fit a list of indicators of successful scale. No second step was required as the transcripts were subject to content analysis immediately after data collection and at the time of manuscript preparation.

## 3 Results

### 3.1 Question 1: design feature enablers and barriers to scale

Design features of the Innovations (i.e., the parenting programs) are presented in Table 3. Three components distinguish the programs, namely their curriculum, the status and training of the providers, and the dosage. The curriculum varied anywhere from 4 to 9 modules addressing how to provide responsive stimulation through play and communication to young children. Behavior change techniques varied: some programs provided visual material to be discussed, such as the counseling cards of CCD in Zambia, and brochures to take home (C4CD+), while others emphasized a demonstration of play followed by parents practicing with their child and receiving coaching (C4CD+; SM). The provision of visual materials to caregivers was thought to be easier to scale horizontally (CCD; C4CD+) than a lengthier demonstration by a provider followed by coaching the practice of a caregiver (SM). Providers also varied from professionals to volunteers. Professionals in a health system were seen to enable vertical and horizontal scale compared to volunteers, in that they were easier to train and retain (C4CD+; Serbia PP). Yet the challenge for professionals was their heavy workload (C4CD+). Given that most of the providers had little experience delivering an early childhood program, they expressed reliance on the manuals provided and appreciated detailed instructions on activities (C4CD+; SM). The resource teams in Bhutan and Rwanda had developed or adapted their own manualized curriculum for providers, whereas the team in Serbia let nurse providers write a set of regulations to guide delivery. The Zambian resource team used an existing curriculum with seven counseling cards that were not yet translated (CCD). Resource teams who had conducted a pilot evaluation were able to make adjustments to improve effectiveness and attendance and convince government stakeholders that the program was beneficial (C4CD+; SM). Dosage varied with some offering 12 weekly sessions and others only 4 over infancy. Frequent home visits were found to be more difficult to scale horizontally in Zambia compared to the group sessions implemented in Bhutan (CCD; C4CD+). Many of these design features determined the effectiveness of the program and therefore indirectly affected scale; their direct effects were on horizontal scaling.

**Table 3:** Design features of programs.

Design features	Bhutan	Rwanda	Serbia	Zambia
Resource Team	Save the Children	Boston College	UNICEF	UNICEF
Program (curriculum)	Prescription to Play (C4CD+)	Sugira Muryango (SM)	Playful Parenting (PP)	Care for Child’s Healthy Growth & Development (CCD)
Amount of stimulation content	9 sessions	4 visits; plus 15 min in all 12 visits	7 infancy visits	7 home visits or 4 group sessions
Other messages	Hygiene, nutrition, discipline	Family conflict; stress reduction	Newborn feeding, hygiene	Nutrition, health, illness
Behavior change techniques	Visual material, demonstration, practice, coaching	Demonstration, practice, coaching	Demonstration, practice, coaching	Visual material, practice
Pilot conducted	Yes	Yes	No	No
Provider of service	Health assistant	IZU (friend of families)	Community nurse	Community-based volunteer
Professional status	Professional	Volunteer	Professional	Volunteer
Education level	Post-secondary	Primary or secondary	Post-secondary	Primary or secondary
Provider manual	Yes, session-specific activities	Yes, session-specific activities	Guidelines	7 counseling cards
Training, refresher	11–12 days, No refresher	10 days, Refresher	6 days Refresher	5 days, No refresher
Dosage, Frequency				
Mode of delivery	Group	Home visit	Home visit	Home, Added Group
Number of families currently seen	9	4	10	10
Frequency	Monthly	Weekly	Irregular, mainly with newborn	1 to 3 months apart during infancy
Number of contacts	12, reduced to 9	12	7	7, reduced to 4

### 3.2 Question 2. What implementation processes were used to expand horizontal reach: What were the enablers and barriers expressed by the resource team, the workforce and government stakeholders?

Findings around horizontal scale refer to the geographic reach of the program, the families within those regions who reported receiving the program messages from a service provider, as well as the number of providers trained and supervised. We present the initial implementation goals of the resource teams and to what extent this was reached 4 years later. Also relevant were the enablers and barriers to reaching horizontal scale. This included evolving plans to train service providers, considerations of the modality used to reach families (e.g., home visits, group sessions, clinic visits), and raising demand at the community level. This section is organized by country, addressing first the level of horizontal scale planned and reached, followed by enablers and barriers as reported by the resource team, workforce and government stakeholders.

#### 3.2.1 Horizontal scale in Bhutan: level, enablers, barriers

In Bhutan, the plan was to implement the program by phase, starting with five districts and expanding eventually to 15 new districts; the latter was done at once because delays caused by COVID-19 meant that scaling had to be speeded up. The initial proposal was to have the Khesar Gyalpo University of Medical Sciences of Bhutan train 639 Health Assistants to deliver the program at 264 clinics and 551 outreach sites where 96% of children under-5 were registered and followed. Therefore, the projected number of beneficiaries was parents of 56,464 children 0–36 months in 20 districts. A pre-pilot evaluation showed effectiveness in child and parent outcomes (31).

Reach was, however, lower than expected. In the last assessment only 30% of eligible families in the original five districts had attended a program session, whereas 50% of families in the scaled-up districts had attended. Still, this meant that 50% had not attended one session out of 12 and only 30% had attended four or more sessions. Reasons for low attendance included distances that families had to walk to reach the clinic, families often forgot when the next monthly meeting was to occur, and an unsystematic method of inviting eligible families.

Enablers. Some enablers of horizontal scale mentioned by the health assistants included feeling respected and appreciated by parents (97% reported this). Also, the fact that pre-service training of health assistants on the curriculum had been institutionalized within a very supportive Medical Faculty meant that new cohorts of 15 providers would be continually available. Most national and regional government stakeholders believed that there was strong demand for the program within the government, but that more community mobilization was required to raise demand among parents and leaders: “*Even in rural areas, community mobilization may not have been adequate to raise awareness or demand*” [stakeholder]. Consistency of delivery and improvements in the quality of delivery were attributed by health assistants to the structured manual of activities they were trained to use in group sessions: “*It helps to guide and refer to before the sessions on what is to be taught to the parents. The second version of the manual is good*” [health assistant].

Barriers. Barriers to increasing horizontal coverage included health assistants feeling overworked with all their clinic duties: *“I like the fact that children and families are benefiting from the sessions, but what would motivate me more is having more staff. There are several programs running alongside as well as reports that need to be compiled. Sometimes, being alone, I get demotivated that I have to do so many different programs as well as make the reports”* [health assistant]. There were plans to ameliorate their workload by training a lower-status cohort of clinic caretakers to assist in the program delivery: “*there are plans to engage clinic caretakers who are paid staff and mostly native to their locality, to raise awareness and attendance at group sessions”* [stakeholder]. Reducing each group session from 90 to 45 min and the number of sessions from 12 to 9 were also suggested solutions to reduce providers’ workload and increase attendance; this was done in the final year of the program. Health assistants also believed that their delivery would improve if they had refresher courses and supervision. Going forward, the plan was to have district medical officers monitor and supervise health assistants. Providers also suggested that an incentive be provided to families to attend, as previously done successfully to boost immunization.

#### 3.2.2 Horizontal scale in Rwanda: level, enablers, barriers

The initial plan for Rwanda was to implement the program in three districts targeting 10,000 children ages 0 to 36 months and both parents. The program was targeted at the poorest 16% of families, who were also included in the government’s cash for work program; thus about 2 or 3 families per village were expected to be eligible. The workforce called *Inshuti z’Umuryango* (IZU), or Friends of the Family, were engaged in child protection activities more widely under the Ministry for Gender and Family Promotion. Two IZUs per village, sometimes covering two villages, were assigned to the program, resulting in 2500 IZUs being trained for three districts. Preliminary evaluations of the program found strong effects on parental practices and some effects on child development (32).

Enablers. Enablers of horizontal scale included the low caseload of IZUs (2.2 families per week), though as volunteers 69% took on other work for on average 28 h weekly. IZUs commended the training and regular supervision they received, and they met frequently with other IZUs to discuss their work and solve problems. In addition, each had a manual covering activities for each session that was highly appreciated as a guide for their delivery. They reported a strong need and a demand for the program that would support scaling to other locations and other status groups: “*parents expressed their request that SM widen its target to people in other strata. This time, when they resume, they should target all citizens”* [IZU].

Barriers. A barrier to horizontal scale was its limitation to the lowest economic status group, and to three districts, though a fourth was being contemplated. Although IZUs expected fathers to attend home visits, only 53% of IZUs reported that fathers were present. A serious barrier was the lack of continued support offered to IZUs once the program finished in their village. It was left to individual IZUs to continue meeting families and only 28% reported doing so: “*The motivation to continue implementing program activities will now depend on personal efforts by authorities in place and not on a formal level of accountability”* [stakeholder]. Stakeholders felt there should have been a clearer exit plan to sustain the program in the districts where it started and expand it to additional villages and eligible families.

#### 3.2.3 Horizontal scale in Serbia: level, enablers, barriers

The initial plan of UNICEF Serbia was to pilot the program in six municipalities and subsequently expand to 27 more (the number was increased to 28 during the second phase of implementation), covering 20% of the country’s municipalities. The long-term goal was to implement in all 158 municipalities. The first group of coordinators and providers helped to train those in the new 28 ones. The initial target to train 750 health professionals, most being home-visiting nurses, was met but was insufficient to serve eligible families. It was expected that 60,000 families would receive the program. The scaled-up municipalities were required to apply for the program, with the intention to have the more motivated and resourceful ones being accepted. A survey of eligible caregivers revealed that 60% had received a home visit but only 32% of these (20% of those surveyed) received messages on child development, and fewer heard about play and communication. A pre-pilot evaluation of an earlier home-visiting nurse program found no effects of the program on parenting practices of non-Roma caregivers (33) and no pilot evaluation was conducted of the current program.

Enablers. The reach and retention of a workforce was less frequently mentioned in Serbia than in other countries. Rather enablers of horizontal scale included the desire of municipalities to adopt the parenting program and take ownership by submitting the required application identifying resources and capacities. Additionally, municipalities who sought to deliver it received support in the form of resources and training by the Standing Conference of Towns and Municipalities and by those who had experience implementing it. Thus, horizontal scale occurred organically with cascading support: “N*ow in meetings when our colleagues from [pilot] municipalities come and start telling other local communities that are just starting to get into all of this, and when they see how nicely everything is developing now, they start wishing for this to begin happening in their local communities”* [stakeholder].

Barriers. Barriers to horizontal scale resided primarily in municipalities and towns that were unwilling or not resourceful enough to submit a strong application showing that they had the capacity and resources to implement the program. Roma families, an ethnic minority in Serbia often living in separate communities, were not sufficiently targeted by the program: “*a Roma coordinator was not included in the coordinating body from the outset, who could have helped involve a greater number of these families in the activities carried out as part of the program. In some municipalities, reaching the most remote families in rural areas has been recognized as a need; in some municipalities, the need. has not been recognized*” [stakeholder]. Even when municipalities adopted the program, they often lacked the personnel, particularly home visiting nurses, to reach eligible families. It was also optional for nurses to undergo training on the program; so far, the numbers were insufficient to reach expected coverage. Of those nurses who did deliver the program, 43% felt overworked and typically saw 10 families per week. Changes to the basic medical program required legislation, for example to provide more visits later in infancy and to require nurses to deliver the play messages.

#### 3.2.4 Horizontal scale in Zambia: level, enablers, barriers

In Zambia, the initial plan was to implement the program in two districts in the Eastern Province. The plan was to train 670 volunteer providers, 350 health facility personnel, and reach 50,000 families with children 0 to 5 years, though the delivery materials applied to children 0 to 3 years. No pilot evaluation of the program was conducted in Zambia ahead of the scale-up. Current coverage, according to independent surveys of randomly selected eligible families, was 36%. Some 1,000 volunteers were trained to deliver sessions.

Enablers. Some of the enablers of horizontal scale, according to the workforce, were their perception of increasing demand among communities and families who saw the benefits, and the support of traditional village leaders. They were supported by monthly meetings with other providers who helped overcome challenges and correct misunderstandings: *“When we meet as a group with my peers, every person will at least give ideas he/she has; then even the challenges we discuss, you can ask questions about things that you do not understand”* [CBV interview]. The resource team was trying to retain volunteers by offering compensation (97% of providers wanted compensation), but this required a government registry that was slow to become fully functional. The main course-correction in the final year was to increase horizontal scale in the two designated districts by adding a group mode of delivery; group sessions were offered mainly at health facilities during weekly growth monitoring and immunization days while parents waited: “*We concentrated on one-on-one or individual counselling, and currently we are doing more of the group counselling*” [stakeholder]. Despite problems in training and retaining a volunteer workforce, most stakeholders felt the Community-Based Volunteer workforce had a solid and sustainable foundation in the community.

Barriers. For several reasons, coverage was not expanding as quickly as expected. Providers stated that the program was not adapted to the dosage required by families, many of whom required more than the allotted home visits and more than the available counseling cards. The workforce and some stakeholders agreed that seven monthly visits were insufficient to change parental practices. The workforce was not sufficiently trained: training was costly and so new cohorts were trained infrequently; only 5 days were given for training and no refresher course offered. Stakeholders agreed that not enough providers were trained; although it was the projected number required, planning was inadequate to meet the intended scale. Government stakeholders and the resource team acknowledged that they were slow to provide compensation and supervision to the workforce, and slow at translating to local languages the materials used to deliver messages. Workforce attrition due to a lack of financial incentive was also reported. The workforce therefore split their time among several organizations (69% did so); as such, some had received training to deliver program content that overlapped with the CCD parenting program, for example, on nutrition and health.

### 3.3 Question 3. What processes were used to scale vertically from community to government: What were the enablers and barriers expressed by the resource team and government stakeholders?

The findings that pertain to vertical scale refer to the extent to which the program had been integrated into the government health system in preparation for a transfer from the resource team to the government. We present the initial goals of the resource team and to what extent they were reached 4 years later. Also relevant were the enablers and barriers to reaching vertical scale. Resource teams and government stakeholders commented on policy and finance, engagement of multisectoral stakeholders in decision-making, and integration of training and supervision of the workforce. Also relevant was an information management system to track workforce and family participation. This section is organized by country, addressing first the level of vertical scale reached, followed by enablers and barriers as reported by the resource team and government stakeholders.

#### 3.3.1 Vertical scale in Bhutan: level, enablers, barriers

The C4CD+ Save the Children team in Bhutan worked closely with the Ministry of Health’s Public Health Division from the beginning, holding regular meetings and conducting advocacy. The Ministry decided at the end to transfer the program from Public Health to the Non-Communicable Disease Division to be overseen by clinical Medical Officers. Another strong partner was the Khesar Gyalpo University who took over the task of training health assistants and then, additionally, medical officers who were to supervise the health assistants delivering the program. Health assistants were professionals within the Ministry of Health, whose duties were tracked in the government’s information system that noted the delivery of the program but not details about which families attended.

Enablers. Enablers of vertical scale in Bhutan included the desire and political will of the government and Ministry of Health to take over implementation of the program. Government stakeholders expressed an eagerness to own the program, to sustain and expand it. The resource team met regularly with government stakeholders to keep them abreast of the horizontal scale-up, and solve issues such as reducing the providers’ workload. The government already was developing and employing an information system that could include information about when providers held their parenting sessions, and could eventually accommodate information about the program participants. Stakeholders from the Medical Faculty of the University strongly supported the parenting program and became advocates for it in communicating with the government. Pre-service training of new cohorts of health assistants was institutionalized within the Khesar Gyalpo University and this was reported to help sustain the program. National and regional stakeholders felt strong ownership of the program, given their involvement in the design and implementation of the program from the start. Although UNICEF had also implemented some parenting sessions, it did not appear to compete with the C4CD+ program of Save the Children.

Barriers. Barriers to vertical scale appeared to be the shift to a new division within the Ministry of Health, with clinical medical officers rather than district health officers in charge of supervising the health assistant workforce. It was not yet known whether this change would proceed smoothly or not. Supervision and monitoring of health assistants delivering the program was minimal throughout the implementation period. Maintaining quality under a new division might be a challenge: “*There is no monitoring that is being done to see how and if we are implementing what we learned to increase attendance or not”* [stakeholder]. With this change in the division responsible for managing the program, capacity building and advocacy were stepped up. Advocacy for the program was still mainly done by the resource team, rather than government agencies, who promoted parenting in a more general way. More multisectoral collaboration was required to incorporate other ministries, such as the Ministry of Education who wanted to include parenting sessions in their well-attended preschool program, and the Ministry of Finance who requested costing information. A government budget was not yet assured; national stakeholders reported that outside funds would be sought.

#### 3.3.2 Vertical scale in Rwanda: level, enablers, barriers

The main government partner of the Sugira Muryango program was the National Child Development Agency who were responsible for coordinating programs of all early childhood health and education partners. The Ministry for Gender and Family Promotion was also an important partner as the agency overseeing the IZU volunteer workforce delivering the program. The initial program design did not integrate explicit activities or structures to sustain program delivery in communities once the program finished its 6-month duration; after their contract terminated, the IZU workforce no longer received training or supervision. Some 72% of IZUs reported that they stopped delivering parenting messages when program implementation ended. Multisectoral committees were set up in districts to meet regularly for comprehensive review of issues affecting families with young children, yet stakeholders indicated that their work was focused mainly on resolving individual family issues. Once financial support from the resource team terminated, local stakeholder engagement waned. There had been no indication of policy or budget changes within the government ministries to integrate and sustain the program.

Enablers. Enablers of vertical scale came mainly from the government prioritizing early childhood education and parenting throughout the country, using community health workers and home-based preschools. Stakeholders noted that “*children’s welfare is included in the performance contract that district officers must fulfill, and that the program’s focus on child nutrition and growth complements government messaging*” [stakeholder]. Multisectoral collaboration teams were in place to continue supporting the program but were not sustained.

Barriers. The main barrier to vertical scale appeared to be the lack of structures and activities to sustain the program within the system. The workforce’s delivery of the program in a village terminated when their contract was over after 6 months. Despite positive feedback on the quality of the program, the government did not take ownership of it and did not indicate that integration of an incentive and supervision would be feasible within existing agencies. No information system was in place to maintain accountability of the volunteers or staff. The only source of financial support for the program was additional donor funding. Within this context, scale and sustainability could be facilitated only if implementers collaborated with other organizations doing similar activities.

#### 3.3.3 Vertical scale in Serbia: level, enablers, barriers

The main government partners of the UNICEF Playful Parenting program were the Ministry of Health with its multisectoral Steering Committee, and the Standing Conference of Towns and Municipalities at the municipal level. The Ministry of Health endorsed legislation given to it by other institutions such as the Professional Guidance for Home Visiting Nurses created by the City Institute of Public Health. A two-year work plan was outlined with deadlines and budget for activities to scale the program. The Standing Conference of Towns and Municipalities coordinated the implementation of the parenting program in the 34 municipalities where it currently existed; it created documents on how to manage the program, plan a budget, seek resources, legislate changes through local governments, and train workers; these documents were available to all municipalities approved to implement the program. Each implementing municipality had its own multisectoral committee to manage services and allocate budgets. Medical schools were integrating messages about child development into their curricula for all health workers.

Enablers. Enablers of vertical scale included the political will and constellating force of the Standing Conference of Towns and Municipalities who coordinated local governments to adopt and implement the program. Although a prior nursing program existed, the Standing Conference helped local governments go beyond medical services for young children and provide an integrated health and development service to families. Local municipalities were eager to apply for the program and to receive help from the Standing Conference of Towns and Municipalities on how to implement the program. Some were receiving funds: “*16 localities were granted financial support from the Ministry of Family Welfare to start up the program in their location*” [resource team]. Various service groups and institutions were eager to collaborate with each other to avoid duplication and provide a streamlined integrated service to families. The National Ministry of Health set up a Steering Committee that could be persuaded to propose the necessary legislation. This legislation was said to be necessary to sustain the program, but in the meantime the Standing Conference and Public Health Institutes were central to creating resources and activities that guided program implementation.

Barriers. Barriers to vertical scale included the need to put all aspects of the program into legislation including new Guidelines for home visiting nurses outlining their duties. As one Ministry of Health (MoH) stakeholder stated: *“We have begun initiatives to put it in the form of a law”* [stakeholder]. This might have helped to overcome a general lack of professional nurses who opted to provide the program. Similarly, a framework for supportive supervision of nurses was not yet in place. A setback in passing new legislation occurred after an election; advocacy with the new government staff was augmented. National stakeholders felt that they were not involved in the planning phase with the resource team: “*Talking about international organizations, there definitely needs to be closer connections and timely collaboration when it comes to such projects, in the planning phase of the project with the MoH”* [stakeholder]. Collaboration was not always forthcoming as institutions changed slowly: “*Essentially, our systems are not interconnected; everyone looks at it from their own perspective. Collaboration is difficult”* [stakeholder].

The decentralized nature of the program, with each municipality free to propose its own approaches and activities served as an enabler of horizontal scale but also a barrier to vertical scale, making it difficult to elevate and institutionalize an approach within the national policy framework, or a set of expectations and standards for the types of services caregivers should receive to support early stimulation and play.

#### 3.3.4 Vertical scale in Zambia: level, enablers, barriers

The resource team, UNICEF Zambia, worked directly with the Ministry of Health, the district focal persons, and its multisectoral committee that included among others the Ministry of Education, Ministry of Community Development, and Ministry of Finance. A framework for a national multisectoral early childhood development policy had been developed; advocacy was ongoing within the parliamentary system to adopt the policy. Multisectoral committees also existed at the provincial and district levels to make decisions about implementing the program. Indicators of program output were developed by a multisectoral team to guide the Ministry of Finance budget allocations. However, funds had to be sought from external sources once the UNICEF resource team departed. The workforce was part of the health system but so far unpaid and not fully integrated. An information system to keep track of service delivery was active at the district level; tracking beneficiaries was still not available.

Enablers. Enablers of vertical scale included the availability of a local volunteer workforce, the Community-Based Volunteers, who already delivered other programs related to health and nutrition. Local civil society organizations had the capacity and were initially engaged to train volunteers on the parenting curriculum selected by UNICEF. After several years, district health officers were put in charge of training. A nursing curriculum on early child development allowed professional health workers to become experts and supervise the workforce.

Barriers. Barriers to vertical scale included a gap between activities at the national level, such as developing an adapted effective curriculum for parenting along with an information system and registry for providers, and implementing those components within communities. The curriculum, the information system and the registry for volunteers were not functioning well. Stakeholders reported that despite pockets of excellence, overall, the quality of service delivery within communities was often inadequate; and onsite supervision and monitoring of the volunteers remained insufficient at the district level. Monitoring forms available in district offices were not used to evaluate delivery and provide feedback. As one stakeholder put it, “*If there are gaps [in delivery], then we need to advocate more to make sure that they [CBVs] also know what they are supposed to do”* [stakeholder]. Current funding was to be tied to output indicators but the government needed to find external funds for the program. Many stakeholders expressed doubts about the government’s readiness to absorb the program financially.

### 3.4 Final year indicators of scale

As funding from external donors was finishing, the level of scale achieved was documented through semi-structured interviews with a small number of government stakeholders in each country. Questions referring to horizontal scale asked about the extent to which the reach of the program had expanded along with training of providers to deliver the program (9). Questions referring to vertical scale asked specifically about the government’s ownership of the program and integration into its health system, about policy and financial support, and inclusion of multiple sectors. Answers to these questions and frameworks from recent publications [e.g., (8, 9, 12)] led to the identification of four indicators of successful horizontal scale (Demand, Reach, Equity, Workforce) and four indicators of successful vertical scale (Multisectorality, Adoption, Policy & Finance, System Integration). Brief definitions are provided in Table 4. Quotes from stakeholders were used to identify the extent to which each program had achieved scale (see Table 4).

**Table 4:** Definitions and quotations reflecting indicators of successful scale.

	Description
Horizontal scale	
Demand	Evidence through attendance and advocacy that families want the program.
Reach	Continuous expansion to new districts and to all eligible families.
Equity	All eligible families, particularly vulnerable ones, have access.
Workforce	A sufficient number of providers are being trained, retained and supervised.
Vertical scale	
Multisectorality	Stakeholders from many organizations and ministries are engaged.
Adoption	Government is willing and able to adopt and implement the program.
Policy, Finance	Government has sustained the program with policy and finances.
Integrated system	Workforce is integrated into the health system and its information system.

Bhutan C4CD+	Evidence for successful scale
Horizontal scaling	
Demand	*We are still trying to figure out the best ways to address [low] attendance. We’ve made it compulsory for health workers to report and include attendance as a performance indicator in their work plans.*
Reach	*Primary healthcare centers and outreach centers are definitely on board, and we plan further expansion based on this strategy. Over the next few weeks and months we will continue to see expansion into hospitals.*
Equity	No quote or comment.
Workforce	*Each year there will be a new batch of graduates and they will apply the sessions in their new community job placement. If we keep involving enthusiastic faculty members every year, we can ensure the continuity and success of teaching the workforce. Because of fund constraints, all onsite monitoring was discontinued.*
Vertical scaling	
Multisectorality	*We have discussed with the MoH to include the university as part of in-service monitoring and supervision.*
Adoption	The MoH adopted the program; it is managed by the Department of Non-Communicable Diseases.
Policy, Budget	*We highly depend on external resources. We can secure more funds from different development partners. We have included this program in our 13^th^ Five Year Plan and this makes it easier to convince all the donors. An executive order from the MoH Secretary guarantees that HAs will conduct the group sessions.*
Integrated in system	*These indicators are mandatory for health workers to write in the information system. Their work responsibilities as outlined in their individual work plan have to be completed for each month and recorded.*
Rwanda Sugira Muryango	Reflective quotes and comments
Horizontal scaling	
Demand	*The expansion of SM that I have seen today is related to families who lived in conflict. Today, they can stand in public and testify how they no longer have family conflict, raise awareness, and always talk about the benefits of the SM program. SM has played a key role in the well-being of citizens.*
Reach	*We have started identifying new families but I would not say we have enrolled them. No new expansion but the government should continue to work with the same families so there is no backsliding.*
Equity	*Families have been helped, especially vulnerable families [the lowest economic strata were targeted].*
Workforce	*We meet IZUs once in 6 months but these meetings are not like the typical trainings done by SM. We want to ensure they understand that even if SM has closed, some activities will have to be implemented without [the Resource Team]. We cannot afford to implement activities as they were implemented by SM as a project.*
Vertical scaling	
Multisectorality	*Collaboration among partners is challenging. We do not have any partners now. Previously they had partners but each had their own scope of intervention. If you see a partner coming to the District, it means that the central authorities have granted access. Families are encouraged to join Saving and Loans groups.*
Adoption	*The government cannot allocate a lot of time and resources the way the project did. I do not think we have done tangible structured activities as a District to scale up the program. SM alerted us they would be closing and we would take up some activities. They gave us resources like training manuals.*
Policy, Budget	*We have been weaned fast because we cannot be self-supportive in such a short time. We do not have the budget. The district did not budget for this activity and would not be able to pay for this activity like onboarding new staff. The district does not have money to facilitate visits, training, and other related activities like essential communication.*
Integrated in system	*Still, we have enormous capacity and logistics gaps. The project removed their staff at the district, sector and cell level.*
Serbia playful parenting	Reflective quotes and comments
Horizontal scaling	
Demand	*We have a lot of new municipalities who want to initiate the program. Parents have the option to choose which professionals they want to hear from.*
Reach	*It is clear that we have reached the target population that we wanted to reach. Through intermunicipal cooperation, smaller communities can unite and act together, to map out resources.*
Equity	*We rejected about 20 local governments in a public call [to adopt the program] because they have no resources; they need help the most. Coverage remains particularly low in rural areas, due to a lack of funds and qualified staff. Single parents attend less frequently. Additionally, the Roma population participates in lower numbers.*
Workforce	*We need to focus on quality staffing, employing qualified professionals. We do not have tools for monitoring at the local level. The biggest challenge remains the general shortage of staff, which is an issue across all sectors. New nurses learn through practical experience under the mentorship of experienced nurses. We distribute materials but what they read depends on them. Young nurses should be trained by the experts, by professionals as we were.*
Vertical scaling	
Multisectorality	*The Standing Conference of Towns and Municipalities is helping local governments with programming and technical support. Three ministries and their professionals are involved, along with municipalities. We need formal agreements so that institutions can recognize each other and offer mutual support. If local actors aren’t involved in policy-making, a lot of well-written strategies end up being impossible to implement. There is need for more intensive intersectoral cooperation among the three systems – health, social welfare, and education.*
Adoption	*There is no strategy at the national level and that dictates everything local governments should do.*
Policy, Budget	*Healthcare is funded centrally at the national level. We have not received any financial support from the MoH. Local governments allocated X million dollars from their own budgets… they applied for financial support from the Ministry of Family welfare and demography who provided X million dollars.*
Integrated in system	*Integrating these programs into regular activities and services within existing institutions, that’s something we are really lacking.*
Zambia CCD	Reflective quotes and comments
Horizontal scaling	
Demand	*The centers are slowly drawing more crowds because communities are beginning to see the benefits.*
Reach	*The numbers are increasing.the numbers are reached through group counseling. We are operating in the same facilities but trying to increase the families at those sites. Two more hubs have been built in a third district.*
Equity	*CBVs work within the areas where they can easily reach without having any challenges with transport. The mothers that show little interest, those that start to fall away. They are followed up by the community.*
Workforce	*We are not training any more CBVs due to finances. We still have faced a challenge of insufficient materials and also issues to do with incentives for the CBVs. Supportive supervision comes only once in a while; the finances are not enough. We have not heard of any CBV getting paid.*
Vertical scaling	
Multisectorality	*Five key line ministries are owning this program … Every time a new government is elected, we do continuous capacity building. But at the district level, all sectors are covered by the district commissioner.*
Adoption	*Yes, we have actually owned the programme. the government has already taken over activities.*
Policy, Budget	*The program is embedded in policy documents in the National Health Strategic Plan 2022–2026. It has got strategic actions and also the targets that we have set for this year. The budget shows commitment… but there are so many competing priorities. We do not have a budget to cover our activities in the district.*
Integrated in system	*It is a reportable intervention to be reported to the Ministry of Finance. There were three output indicators but recently these have been revised. They have been slow to implement it.*

Sources of quotes were omitted to preserve anonymity.

Bhutan’s government had fully adopted the C4CD+ parenting program. Regarding horizontal scale, they have implemented the program in Primary Health Centers and outreach sites of all districts, however, low attendance (low demand) was still a problem. They were starting to implement in major hospitals around the country. The medical faculty had institutionalized training of a cadre of health assistants each year but supervision was still lacking. Regarding vertical scale, the government was now fully in charge, but the program was moved to a new division within the Ministry of Health. The Medical Faculty was firmly involved but other ministries such as education were not yet engaged, despite their presence in each village in the form of preschool centers. Health assistants who provided the service were required to record their program delivery as part of their regular duties. The main limitation was lack of funds, though stakeholders were confident that development partners would support them.

Rwanda’s national government had no intention of adopting the Sugira Muryango program. Sustainability was not assured as it depended entirely on whether the volunteer providers would continue to deliver messages along with their other child protection duties. This appeared unlikely without donor support. The most vulnerable families were targeted by the program (16% of the population) but no new families were being enrolled. Regarding vertical scale, the lack of national and district government support and the lack of sustained multisectoral partners meant that the program would likely terminate unless a new short-term donor appeared. The main government body, namely the National Child Development Agency, served to coordinate but not implement programs. They lacked policy, budget and human resource capacity.

Serbia’s national government was reluctantly engaged. However, stakeholders from local governments and the Standing Conference of Towns and Municipalities had adopted the Playful Parenting Program. Demand for the program was strong among municipalities. Reach was expanding rapidly among them. However, equity was a problem in that the applications of municipalities with low resources were rejected; rural populations and Roma communities were less likely to receive the service. The availability of home-visiting nurses was a major constraint and their training was not systematic. Regarding vertical scale, municipalities and the coordinating Standing Conference were taking ownership. The lack of engagement by the Ministry of Health was seen as a major impediment to coordination, policy development, legislation, and financial support. However, other organizations and ministries such as Welfare and Education were sometimes filling the gap. Stakeholders were concerned about the lack of policy and legislation to regulate how the service was coordinated and delivered.

Zambia’s Care for Child’s Healthy Growth and Development program was adopted by the Ministry of Health. They noticed heightened demand at centers, and reach had been expanded in the past year due to the delivery of group sessions at facilities and community hubs, along with some home visits. Outlying rural areas were unlikely to be serviced due to transportation challenges. Training of new volunteer providers had stopped due to lack of finances, and onsite supervision was lacking. Although a registry to manage stipends was now available, volunteers had not yet benefited from it. Regarding vertical scale, multisectoral support from several ministries was present at the national and district levels. A policy was adopted but changes in government personnel meant that advocacy among ministries had to be re-initiated. Service delivery was being inserted into the health information system. As with other countries, financial support was not assured.

## 4 Discussion

Findings from this study address critical features of scaling parenting programs raised in previous publications [e.g., (8, 12, 27)], specifically as they pertain to these four implemented programs. The study also provides novel insights about vertical scale, a dimension that has not been previously explored in detail. As with previous implementation frameworks used to evaluate scale in parenting programs (8, 12), we focused on enablers and barriers to scale, including features of the innovations (intervention) and the implementation process, from the perspective of implementers (resource team), workforce, and government end-users. In brief, enablers and barriers to horizontal scale were often connected to the workforce and to community demand for parenting support. Enablers and barriers to vertical scale were more likely to be connected to political will and capacity within the government for policy development and funding, to multisectoral collaboration, and to the human resources and information tracking of the system. Finally, we proposed eight indicators of successful scale that may be used by governments, organizations, and donors to monitor their achievement at scaling a program. Findings are now interpreted as they apply to the two dimensions of scale.

### 4.1 Horizontal scale

Of the six building blocks for scalable parenting programs proposed by Buccini et al. (12), one concerns the intervention design features and three concern the workforce. This emphasis aligns with enablers and barriers to horizontal scale found in the four parenting programs described here. First, we address intervention design features, such as the curriculum, modality and dosage, and how they could determine its effectiveness and demand for the program. To yield effectiveness and demand, the design features needed to be aligned with the experience of the audience, both provider and caregiver. A structured curriculum that elaborated on each activity might be easier for an inexperienced provider, as in the case of Bhutan’s C4CD+ and Rwanda’s SM, and thus improve workforce training and retention as an enabler of horizontal scale. High dosage might hamper horizontal scale but be important for effectiveness with families who were unfamiliar with brain development and the need for early responsive stimulation. According to data from MICS surveys (34) and other reports (31, 35, 36), only Serbian parents in these countries were familiar with the need for early stimulation. Finally, group sessions extended horizontal scale faster than home visits (Bhutan C4CD+; Zambia CCD), and provided their own social benefits, but presented a challenge to attendance (4).

Community demand for the parenting program selected by the resource team was initially low in most cases (15), possibly because parents did not know what they did not know; they tended to hear from health workers about their child’s growth and health but not about mental development. Most parents were unaware that practices related to responsive stimulation, such as the provision of play and conversation with their child from birth, can affect their child’s mental development [e.g., (13)]. Two programs—in Bhutan and Rwanda—collected early pilot data on the effectiveness of their program in improving parenting practices and child development, and thus adapted their curriculum and dosage to the audience. Others—the UNICEF programs in Serbia and Zambia—did not conduct prior evaluations of the current program in their context and later found that the curriculum and dosage may not have met the needs of the population.

Uptake at the community level required a program that was adapted and effective, but it also required effective advocacy within communities targeting community leaders and parents. These three components, namely adaptation to the audience, evidence of effectiveness, and advocacy within communities were mentioned as serious barriers to the Criança Feliz program in Brazil (8). For example, when the problem of finding and enrolling eligible families was noted, efforts were made to increase dissemination of materials and community events to raise the program’s profile. Some families needed convincing to attend the program when they heard that money would not be offered. Moreover, although families and providers of the Brazil program noticed changes in children and parental practices, both commented on the insufficient dosage, content, and quality of delivery (8).

The workforce’s contribution to horizontal scale was critical in the four programs discussed here. Because most health workers had little experience and expertise in early child development, each new cohort of volunteers and professionals required training, monitoring, and supportive supervision, along with a manual of specific activities to guide delivery. A volunteer workforce showing high attrition is a challenge to geographic expansion when they leave for more remunerative work. Community volunteers also often have low levels of education and literacy, thus requiring much more frequent and supportive supervision to ensure quality. This element of supportive supervision—intensive, yet feasible within existing systems—was a serious challenge for the two programs that used volunteers (in Rwanda and Zambia). However, a workforce of health professionals who are overburdened with clinical duties can also challenge horizontal scale.

An alternative not used here is paraprofessionals, such as community health workers who typically focus on preventive and promotive services, such as parenting programs (22, 25). Yet, high turnover, high workload, and low incentives also plagued the Brazilian scaled-up program, where providers unanimously wanted more supportive supervision and some reported that quality was sacrificed for the sake of rapidly increasing coverage; as a result, the government instituted better training, better delivery materials, monitoring checklists and quality indicators (8). These improvements in inputs were aimed at improving not only the quality of service delivery but also the effect of the program on parent and child outcomes.

Fidelity and quality of delivery were mentioned as issues in Bhutan and Zambia but were critical in all settings. Fidelity refers to delivering the curriculum as intended. This was more easily accomplished in sites where a detailed manual of activities was provided (C4CD+, SM, and CCD). However, fidelity to a weak curriculum does not meet quality standards. The activities must include techniques to change parenting practices. Additionally, the workforce requires training, refresher courses and supportive supervision from an integrated health system. As such, achieving quality delivery overlaps processes related to horizontal and vertical scale. Two publications outline how monitoring the quality of delivery in these programs yielded useful feedback for improvement (2, 3).

Most of our four programs encountered provider shortages, either because new cohorts were not trained or they received inadequate incentives (Zambia CCD) or because professional training in the program was optional (Serbia PP). The providers in all four programs reported feeling respected by parents, but were not well monitored or supervised in three of the programs—the fourth, in Rwanda, provided strong monitoring and supervision but it was offered by paid staff rather than government supervisors and so not sustainable. Supportive supervision of providers is necessary to maintain demand for a high-quality program (see (18)). Decisions about the workforce were often made by the resource team ahead of implementation, and a course-correction was required once its constraints on scale were discovered. To reach eligible families in intended numbers required training an additional cadre of workers (in Bhutan), providing a stipend or other incentive to volunteers (Zambia), and/or using group sessions as a mode of delivery (Zambia; Bhutan always had group sessions). Changes were usually made in the fourth year of operation so it was not yet clear whether the new strategies would solve the early constraints on horizontal scale.

### 4.2 Vertical scale

Less has been written about vertical compared to horizontal scale. Vertical scale refers to policy and program development that aims for sustainable integration within the system. In Buccini’s (12) framework, only “intersectorality” also known as multisectoral collaboration was a building block to support vertical scale. Yet the Brazilian program, despite having strong political support at the national level and partnerships with civil society organizations and academia, sufficient to withstand pandemic constraints and a shift in government, discovered that the program lacked effectiveness in improving parent practices and child development (8).

In our analysis of four parenting programs, enablers and barriers to vertical scale also included political will (“ownership”) and capacity within the government to manage the program, develop policy and funding mechanisms, and integrate information into a health system that tracked human resources and service delivery. Vertical scale strengthens sustainability. Unfortunately, many programs that take a bottom-up approach provide strong evidence of effectiveness at the community level but then lack adoption by regional or national governments; they are not sustained once funding stops (16). Others are adopted from the start by the national or municipal government (top-down approach) but encounter ineffective delivery at the community level because of an intervention not suitable to the audience or the workforce. So, success at vertical scale is not necessarily an assurance of effective outcomes.

In their discussion of multisectoral collaboration, Buccini et al. (12) included features such as multiple ministries (e.g., health and education), multiple organizations (e.g., civil society organizations), and multiple professionals delivering the same parenting messages. This was the case in the top-down approach taken in Zambia where multisectoral committees including stakeholders from four ministries were engaged at the national and district levels, where organizations helped to train providers before passing this over to district-level health officers, and where nurses at health facilities as well as community volunteers delivered messages to families. It was also the case in Serbia where the Ministry of Health along with Institutes of Public Health and the Standing Conference of Towns and Municipalities were involved in creating and passing policy or coordinating implementation. Serbian home-visiting nurses were the main frontline providers visiting families of newborns, but pediatricians, social workers, preschool teachers, and those conducting parenting classes were disseminating messages about responsive stimulation to their clients. Thus, multisectoral collaboration was higher in the top-down approaches used in Serbia and Zambia, but both lacked evidence of sufficient dosage. Consequently, the overall impression was of a diffuse, light-touch program that was not yet reaching intended families. In sum, multisectoral collaboration is a strong enabler of vertical scale but it brings additional challenges. One of these is that multiple light-touch service providers who are not monitored or supervised run the risk of disseminating unsystematic messages about parenting practices and child development.

Other enablers and barriers of vertical scale included the political will and capacity of the end-user, namely the government (e.g., Ministry of Health), to adopt and sustain the program. Political will was strong in Bhutan where the Ministry of Health explicitly expressed a desire to transfer the program from the donor to the Ministry from the start. The Ministry staff felt a strong sense of ownership, relied on a capable medical faculty to train providers, and frequently requested information on cost and impact. Costing studies for individual programs are still rare in this field [but see (14, 37)]. In contrast, the Rwandan government did not have the will or capacity to adopt the SM parenting program; it was satisfied to let the National Child Development Agency coordinate multiple programs implemented by multiple donors without requesting that they collaborate. The Zambian Ministry of Health felt strong ownership and developed a multisectoral policy framework but did not have the funds to train or retain providers. Likewise, the Standing Conference in Serbia had the capacity to coordinate implementation; yet they lacked strong policy, legislative and financial support from the Ministry. All required external financial support and constant advocacy to maintain the programs.

In sum, less has been written about enablers and barriers to vertical scale than horizontal scale. In addition to what is known about multisectoral collaboration, our data revealed that the engagement of multiple sectors brings challenges as well as facilitators. Multiple service providers, some providing an intense universal service and others a targeted light-touch service required coordination, yet the information systems were not sufficiently developed to track services and make them accountable. Vertical scale also depended on political will and capacity to adopt the program, which some programs had. Most, however, were unable to provide the finances to sustain it and so looked externally for funds.

### 4.3 Indicators of successful scale

We proposed eight indicators of successful scale that were relevant for these parenting programs for early child development. They include four for horizontal scale: demand, reach, equity and workforce. The workforce, selected initially, set limits on their reach and most had difficulty training and retaining a sufficient number of providers to expand their reach. Low demand in communities required advocacy, and after a change in government advocacy was required to maintain interest among lawmakers. In Bhutan, reach was high in that it covered all districts, but demand was only moderate as evidenced by low attendance. In contrast, reach was lower in Zambia and Serbia, due mainly to insufficient providers.

Four indicators of successful vertical scale were: multisectorality, adoption by the government, sustainability through policy and financial support, and integration within the system. The parenting programs in Serbia and Zambia had the greatest levels of vertical scale with multisectoral support and adoption by some level of government. Bhutan’s program was eagerly adopted by the government’s Ministry of Health, and sustained by a workforce integrated into the health system. All programs faced financial insecurity, though some governments were more confident than others at proposing solutions to this. It seemed clear by the final months that some programs would continue to scale horizontally and sustain their program over the coming years.

### 4.4 Recommendations

Based on our findings derived from years of research and learning with four different parenting programs, we offer four recommendations for organizations and donors associated with scaling parenting programs:

1 Start building enablers to vertical scale with the relevant government focal ministry from the beginning. This will build political will and capacity. Hold regular meetings to keep them informed, disseminate advocacy, and seek their opinions. Continuous iterations of the program may be required to make it scalable from their perspective.
2 Attend to the design of the program from the start, namely the curriculum, modality, and dosage. They should suit both the providers’ and the community’s understanding of parenting for development. A pilot evaluation is a necessity so that corrections can be made early in the process.
3 Organizations often have little choice over the professional status of the provider recruited to deliver the program. Most providers will have little prior expertise and experience delivering messages about child development and responsive stimulation, so the manualized curriculum should be structured, prescriptive, with lots of examples of responsive stimulation activities, written in the local language. Each status will have its own enablers and challenges, e.g., professionals already have a heavy workload and so will need a cadre of support staff; volunteers need more training, refreshers, and supervision. Both types of providers need incentives.
4 Conduct regular implementation research to provide valuable information on issues such as workforce recruitment and retention, delivery quality, attendance, father engagement, multisectoral stakeholder perspectives, and caregiver enrollment. Timely feedback encourages agile course correction.

### 4.5 Limitations and strengths

Several limitations of the study reduce confidence in our conclusions. Here we collected data at specific times during a four-year scaling process of four countries and collected data from three sources only. Caregivers might have a different perspective, especially on their demand for and benefits from the programs. Our conclusions have limited generalizability to other countries and programs. We used largely qualitative data from semi-structured interviews that allowed for content analysis and generated meaningful quotes. We also used surveys and documentation (e.g., delivery manuals). Quantifying our proposed indicators of successful scale would be a very useful future contribution to the field. A major strength of the study was its independence from the program implementers. The stakeholders from whom we collected data on scale were central to the scaling process and provided confirmation of each others’ views. Useful comparisons could be derived when similarly analyzing four programs. The identified enablers and barriers to scale in four very different parenting programs and the indicators of successful scale are novel contributions that other programs can apply, modify and expand.

## Data Availability

The datasets presented in this article are not readily available due to ethics restrictions. Restrictions apply to interview data that cannot be anonymized due to the ease of identifying the respondent. Some anonymized data may be obtained from the authors. Ethics approval will have to be obtained from the requesting party and from the authors’ ethics board. Requests to access the datasets should be directed to comoeva@fhi360.org.
